# Prediction, Explanation, and Control: The Use of Mental Models in Dynamic Environments

**DOI:** 10.1162/opmi_a_00112

**Published:** 2023-11-27

**Authors:** Roman Tikhonov, Simon DeDeo

**Affiliations:** Department of Social & Decision Sciences, Carnegie Mellon University, Pittsburgh, PA, USA; Santa Fe Institute, Santa Fe, NM, USA

**Keywords:** exploratory learning, finite-state machines, dynamic decision-making, mental models, counterfactual reasoning

## Abstract

The abilities to predict, explain, and control might arise out of operations on a common underlying representation or, conversely, from independent cognitive processes. We developed a novel experimental paradigm to explore how individuals might use probabilistic mental models in these three tasks, under varying levels of complexity and uncertainty. Participants interacted with a simple chatbot defined by a finite-state machine, and were then tested on their ability to predict, explain, and control the chatbot’s responses. When full information was available, performance varied significantly across the tasks, with control proving most robust to increased complexity, and explanation being the most challenging. In the presence of hidden information, however, performance across tasks equalized, and participants demonstrated an alternative neglect bias, *i.e.*, a tendency to ignore less likely possibilities. A second, within-subject experimental design then looked for correlations between abilities. We did not find strong correlations, but the challenges of the task for the subjects limited our statistical power. To understand these effects better, a final experiment investigated the possibility of cross-training, skill transfer, or “zero-shot” performance: how well a participant, explicitly trained on one of the three tasks, could perform on the others without additional training. Here we found strong asymmetries: participants trained to control gained generalizable abilities to both predict and explain, while training on either prediction or explanation did not lead to transfer. This cross-training experiment also revealed correlations in performance; most notably between control and prediction. Our findings highlight the complex role of mental models, in contrast to task-specific heuristics, when information is partially hidden, and suggest new avenues for research into situations where the acquisition of general purpose mental models may provide a unifying explanation for a variety of cognitive abilities.

## INTRODUCTION

We interact with dynamical systems on a daily basis, ranging from simple physical objects like doors or bicycles to more complex ones such as electronic agents and our fellow human beings. To interact successfully requires that we make accurate predictions about their future states, that we reason correctly about the underlying causes, and that our interactions influence their behavior in adaptive ways. In any particular interaction, all three tasks are likely to come into play: when driving on a highway, for example, we may need to *predict* the actions of other drivers, *explain* the causes of unexpected behaviors, and (potentially) attempt to *control* the outcome by honking the horn to avoid an accident.

While a great deal of work has studied human performance on this triad of prediction (e.g., Bates et al., 2015; Uppal et al., 2020), explanation (e.g., Dorfman et al., 2019; Gerstenberg et al., 2021; Wojtowicz & DeDeo, 2020), and control (e.g., Brehmer, 1992; Hundertmark et al., 2015; Osman, 2010), it has tended to focus on one ability in isolation from the others. There is, however, growing evidence that these abilities interact in complex ways. For example, Hagmayer et al. (2010) demonstrated spontaneous causal learning in a dynamical system control task, challenging a common assumption that control relies mainly on procedural knowledge. Similarly, a close interaction between explanation and control has been suggested by Steyvers et al. (2003) and Davis et al. (2018), while Glauert (2009) shows that accurate predictions can be made even in the absence of accurate explanations.

A deeper understanding of any of these three abilities, in other words, seems to require understanding how they interact with each other and how they may, indeed, be built from a common source. One common approach—though not the only one—is to organize these abilities into an ascending hierarchy. Pearl’s influential framework, known as “the ladder of causal inference” (Pearl, 2009; Pearl & Mackenzie, 2018), does this, making sense of the interconnected nature of prediction, control, and explanation as distinct levels of cognitive processing, with each level building upon the capabilities of the previous level, while incorporating additional assumptions.

In Pearl’s picture, prediction (observational queries), at the first level, involves estimating probabilities based on observed associations and requires no interaction with the system (e.g., *How likely is a car accident if one drinks and drives?*). Control (interventions), at the second level, involves causal reasoning about the effects of actions on the system to achieve desired outcomes (e.g., *How can I avoid a car accident?*). Explanation (counterfactual queries), at the highest level, involves estimating probabilities based on counterfactual causal reasoning, *i.e.*, retrospective thinking about scenarios that differ from the actual one (*Would I have been in the car accident if I hadn’t been drinking?*). In this approach, explanation is considered the most challenging task; control is relatively easier because it does not require counterfactual reasoning; and prediction is the least demanding task because it relies on simple associations.

An implicit assumption of this framework, shared by various traditions (Craik, 1967; Griffiths et al., 2010; Johnson-Laird, 2010; Jones et al., 2011; Norman, 1983) is that individuals rely on mental models to accomplish these tasks. A mental model can be seen as a largely tacit but nonetheless systematic representation of the object to hand, incorporating both prior assumptions about the system’s structure, and direct experience, that enables reasoning about alternative outcomes and the adaptive handling of uncertainty. With a complete and accurate mental model, and in the absence of cognitive constraints, individuals should perform equally well in predicting, controlling, and explaining events.

In reality, however, empirical studies show that performance in these tasks can vary significantly. For example, Berry and Broadbent (1984, 1988) demonstrated that people could be equally good at controlling and predicting a dynamic system under a salient rule, but struggled to accurately predict while maintaining control when the pattern became less obvious. Another study (Brown & Steyvers, 2009) found that participants were better at identifying hidden states of a dynamic environment (the explanation task) compared to predicting future states in a normatively identical task. Somewhat similar results were found in Fernbach et al. (2010, 2011) showing that people are better at making diagnostic (i.e., explanatory, backward-reasoning) judgments, compared to predictive, forward-reasoning judgments about the probabilities of future events. They referred to deficits in the forward case as *alternative neglect bias*: a tendency to ignore alternative causes of a given event.

Roughly speaking, there are three ways to explain these emergent differences in performance: through differences in learning, through differences in discrimination and judgment, and through differences in computational load. First, it may be the case that, for commonly encountered dynamical systems, a representation that produces good performance on explanation, say, takes longer to learn than one that produces good performance on prediction or control. One might need to interact with a system for longer in order to gain enough experiences of different alternatives to make explanation possible.

Second, it may be the case that explanatory judgments (say) require more subtle judgments about relative levels of evidence. Even when the representation is optimal, the “average” explanation question for any particular dynamical system may involve comparing possibilities that are almost equivalently good, while the “average” control question may present a clearer, and more decisive, choice.

Third, it may be the case that using the model for different tasks may impose different computational burdens. The underlying structure of the representation may be more easily rearranged to produce an answer to a prediction question, for example. Or, some tasks may require cognitive capacities, such as the ability to project forward or backward in time, or to imagine counterfactual possibilities, that are more demanding or prone to error. Finally, systematic deviations from a normative standard—e.g., neglect of less-likely alternatives, as in alternative neglect bias—may harm performance on one task more than another. While the first two sources of differences in performance have important implications for real-world behavior, it is this third possibility that is perhaps of greatest interest to cognitive science, concerned as it is with the ways in which we make, and make use of, mental representations. Ascending Pearl’s hierarchy, for example, is largely a matter of navigating increasingly complex computational challenges.

This paper presents a novel experimental paradigm that enables us to examine how people predict, explain, and control dynamical systems under different levels of complexity and uncertainty. Our paradigm uses finite-state machines (FSMs) to create dynamical systems that vary in complexity and can introduce uncertainty by hiding some of the information relevant for decision-making. We operationalized *prediction* as the ability to identify the most likely future state of a system, *explanation* as the ability to determine the input that has the strongest causal connection with a given output, and *control* as the ability to manipulate a system’s behavior to achieve a desired outcome.

We conducted four experiments using a *Chatbot Interaction Task*, in which participants interacted with an artificial conversational partner using a limited set of emoji icons. In Experiments 1–3, participants began with a free-exploration phase, followed by test tasks designed to evaluate on their abilities to predict, explain, and control the dynamic system.

Experiments 1 and 2 examined the effects of FSM structure and hidden information on prediction, explanation, and control abilities. After free exploration, each participant was tested on one of the three tasks. We had two main goals in this first experiment. First, to investigate how task performance varied with the presence of hidden information; our hypothesis was that performance would tend to equalize when information was hidden, because participants would have to rely on an underlying mental model, rather than task-specific heuristics (Hypothesis 1). Second, to test for the presence of the alternative neglect bias. As described above, prior work suggests that alternative neglect might apply to explanation more than control or prediction (Hypothesis 2); our paradigm allows for direct comparison between the three tasks.

Experiment 3 further explored the interrelationships among these three skills, employing a within-subject design, testing the hypothesis of a shared underlying factor (i.e., a mental model) across all three abilities. Our hypothesis was that better performance on one task should predict better performance in another (Hypothesis 3). This is because we thought that performance on any particular task would be driven, in part, by the learning and mastery of the underlying mental model, which would give individuals improved performance on the other tasks as well.

Experiment 4 aimed to determine causal relationships among prediction, explanation, and control by evaluating knowledge transfer from one skill to the other two. In contrast to Experiment 3, with a free-exploration phase followed by testing, participants in Experiment 4 were explicitly trained on one task, and tested (without feedback) on two others. This final experiment also introduced an adaptive feedback-based learning method with a predetermined accuracy threshold to address limitations identified in the preceding experiment.

We had two main hypotheses in Experiment 4. First, that there should be skill transfer: participants trained on one task should be able to perform above-chance on other tasks (Hypothesis 4). This is because we believed that task mastery would require the creation of a mental model that could then be used for the other tasks. Second, that we should see correlations between performance on the trained task and the other tasks (Hypothesis 5). This follows the logic of Experiment 3; here, performance on the trained task would be driven, in part, by the creation of the mental model, which would drive performance on the other tasks as well.

## MENTAL MODELS AND DYNAMICAL SYSTEMS

Dynamical systems vary enormously in complexity, from the simplest one-knob thermostat to the rich grammars of the natural languages people use with each other (Crutchfield, 1994); a full accounting for all their varieties falls outside of the scope of this paper. Here, we focus on the (mathematically) simple case of probabilistic finite-state machines (FSMs), which can be understood as an interactive version of a first-order Markov Model. FSMs may be simple, but they are also commonly encountered in day-to-day life. Many simple mechanical and electronic devices are FSMs, and more complex systems can often be well-approximated by them as well.

**Figure 1.**
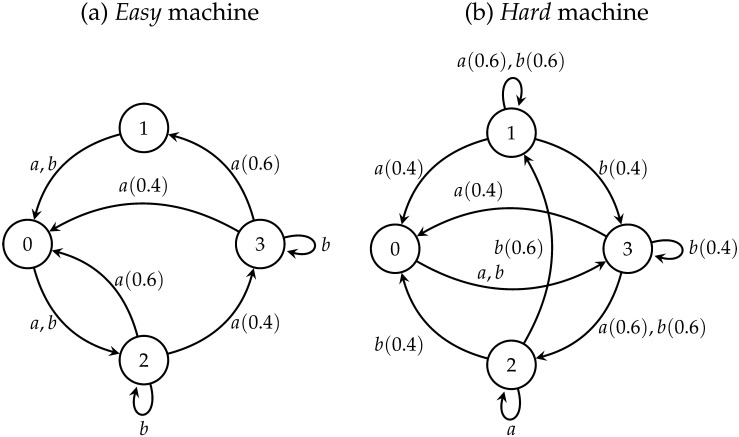
**Finite-state machines that define responses of the *chatbot***. Each of the four states represents an emoji icon sent by the chatbot, while inputs (*a*, *b*) correspond to emojis sent by participants. Parentheses indicate probabilities for inputs with multiple next states.

The two FSMs used in the experiments of this paper are shown in Figure 1. As with any FSM, each has a finite number of internal states (in our cases, four, labeled from zero to three), and a finite number of input symbols (in our cases, two, labeled *a* and *b*). At any point in time, the FSM is in one of its internal states; upon receiving an input symbol, it switches to a new state, following the rule associated with its current state. As an example, consider the “easy” machine of Figure 1. If it is currently in state 2, and receives the input symbol *a*, it switches to state 0 with probability 0.6, and to state 3 with probability 0.4.

Figure 1 defines the FSM in terms of its underlying causal structure, but (of course) this is not how dynamical systems are presented to us; in reality, we simply interact with a system over time, observing the actual outcomes of our interventions as the system changes state. Building on recent work, (Tikhonov et al., 2022), we understand prediction, explanation, and control tasks as relying on an underlying mental model of the system—a largely tacit, implicit, and probabilistic representation of how the system works that attempts to capture the causal structure—that takes a Bayesian form. In our experiments, the idea is that the participant constructs a mental model during the free exploration phase, which is then used to answer the test questions in the second phase.

An individual who possesses a mental model will, in general, have only partial conscious access to its structure and can only articulate some fraction of what it contains. Called upon to predict, explain, or control a system in response to test questions, the individual faces the problem of making some aspects of the model explicit, and capable of guiding deliberative action.

We model this translation of implicit knowledge to explicit decision-making in two stages. In the first stage, we consider the participant’s mental model of the underlying machine. This takes a Bayesian form, specifying the (probabilistic) response of the machine to different inputs: explicitly, the mental model gives the reasoner access to the probability distribution *P*(*i*|*j*_*k*_), where *j* is the current state, *k* is the current input symbol, and *i* is the next state. In the case of the FSM, this model has the same mathematical form as the true structure, making subsequent analysis simpler.

This probabilistic model is then used by the participant to judge the relative likelihoods for the different tasks. The form of the tasks and the normative answers—*i.e.*, the way in which the correct answers are calculated—are shown in Table 1. In the prediction case, for example, the participant is asked to predict the response of the machine to a series of inputs, making a binary choice between final State 1 (say), and final State 2. The mental model provides a degree of belief in these two outcomes, *P*(1), and *P*(2), which can be summarized as the relative log-likelihood, *R*, of the more likely choice; if *P*(1) is larger than *P*(2), this is

$$R=\log\frac{P\left(1\right)+\epsilon }{P\left(2\right)+\epsilon },$$ (1)

where *ϵ* is a small regularizing parameter that takes into account that the participant may attribute some small probability to an outcome that their mental model says is, formally, impossible.

**Table 1.** Normative answers to the test tasks. The probabilities *P* are given by the subject’s mental model, which is learned from free exploration in phase one of the experiment. States are digits from 1 to 4, responses are subscript lowercase letters (*a*, *b*), hidden information is X, and two-alternative forced choice queries are “?” (see also Figure 1).

Basic Form	Question Format	Normative Answer
**Prediction** (*What is the most likely next state?*)	Visible: 1*_a_*2*_b_***?**	argmax*_i_ P*(*i*|2*_b_*)
	Hidden: 1*_a_*X*_b_***?**	argmax*_i_* ($\sum_{k=1}^{N}$ *P*(*i*|*k*_*b*_)*P*(*k*|1*_a_*))
**Explanation** (*Which input caused the system to be in that particular final state?*)	Visible: 1_**a**_2_**b**_3	*P*(3|2*_b_*)*P*(2|1*_b_*) < *P*(3|2*_a_*)*P*(2|1_*a*_)
	Hidden: 1_**a**_X_**b**_3	$\sum_{k=1}^{N}$ *P*(3|*k*_*b*_)*P*(*k*|1*_b_*) < $\sum_{k=1}^{N}$ *P*(3|*k*_*a*_)*P*(*k*|1*_a_*)
**Control** (*Which input(s) will most likely reach the goal state?*)	Visible: 1*_a_*2_**?**_3	argmax*_j_ P*(3|2*_j_*)
	Hidden: 1_**?**_X_**?**_3	argmax_{*i*,*j*}_ ($\sum_{k=1}^{N}$ *P*(3|*k*_*j*_)*P*(*k*|1*_i_*))

*Note*. Here, *N* is the number of intermediate states. In the explanation task example, we show the case where the correct answer is “because the first input was *a*”.

The value of *R* is taken to be more-or-less implicit content, which the participant needs to act on. We assume that this happens in a noisy fashion; if 1 is the correct answer at evidence level *R*, then the participant chooses 1 with probability *p*_*C*_ given by

$$p_{C}=\frac{\exp\left(\beta R\right)}{\exp\left(\beta R\right)+\exp\left(-\beta R\right)},$$ (2)

where *β* parameterizes the noise in the translation from implicit to explicit knowledge. When *β* is large, the participant makes efficient use of the knowledge *R*; when it is small, the choice is much less reliable; when it is equal to zero, the choice is random.

To use this model to understand human decision-making, we first construct an approximation to the mental model that the participant possesses on the basis of their free exploration, based upon a simple frequency-based rule: *P*(*i*|*j*_*k*_) is equal to the number of times the participant observes state *i* following state *j* under input *k*, divided by the number of times they saw state *j* under input *k*. For any question *i*, we then compute the relative probabilities, *R*_*i*_, of the two options for the answer to a task question (see Table 1): in the prediction task, this might be the relative probability of the system ending up in State 1 versus State 2; in the control task, the relative probability of the system ending up in the desired state, given that the agent chooses to do either action *a* or action *b*; in the causal (counterfactual) explanation task, the relative probability that the system would have behaved differently if the first action was changed to the alternative, versus the second action. This is given by Equation 1.

Finally, we see how well the choice indicated by the mental model matches the actual behavior of the participant. Formally, this is simple: the *β* parameter is simply the coefficient in a logistic regression on the correct answer to question *i*, with the independent variable *R*_*i*_, without intercept. By comparing *β* values across different tasks and conditions, we can determine the degree to which people are able to use their mental models to do prediction, explanation, and control.

There are two reasons to go through this somewhat elaborate process. First, different tasks will have different difficulties: a person’s mental model may give clear guidance for a prediction task (i.e., suggest a decisive choice, with large *R*), but a much weaker one for a control task. What we care about is the reliability of the *use* of the model at a fixed level of evidence *R* (given by *β*), not the actual fraction of correct answers, which is a mixture of both *β* and *R*. If we simply score participants on performance, we will confuse tasks that are difficult because the answers are less clear, with tasks that are difficult because participants struggle to use their mental model well. Second, use of the mental model (the *P*(*i*|*j*_*k*_)s built up from experiences during the free exploration phase) rather than the true model (the *P*(*i*|*j*_*k*_)s that correspond to the actual system, *i.e.*, those of Figure 1) enables us to correct for the fact that some aspects of the system, relevant for performance, may be harder to learn than others. For example, if the free exploration phase did not provide enough experiences necessary to distinguish between two options in a particular question, the probabilities will be equal for that participant, and thus the *R* for that participant will be zero, reflecting the absence of evidence for that participant.

An additional—crucial—benefit of our approach is that it allows us to compare different ways a participant might use a mental model to accomplish the task. While Table 1 provides the rules for giving the normative (i.e., Bayesian) answer to the different questions, we also consider an alternative, heuristic approach that participants might fall back on in cases where there is hidden information. This is the alternative neglect (AN) heuristic introduced in Tikhonov et al. (2022) and related to the bias discussed by Fernbach et al. (2010, 2011); in cases where some of the information necessary for the task is hidden, our AN heuristic says that participants do not sum over all possibilities for the hidden information, but instead assume that it takes the maximum likelihood value.

As an example of how the alternative neglect heuristic can differ from the “proper” normative rule, consider the case of the “hard” machine, and the case where the participant is told that the machine begins in State 2, and receives the input *a* twice, and is not told what state the machine is in after receiving the first input. If, in the process of making a prediction, the participant does the normative analysis, their mental model (assuming, for the moment, that it matches the true model closely) will estimate the probability of the final state being State 0 as *P*(0|3*_a_*)*P*(3|2*_a_*), or approximately 0.16. Under alternative neglect, however, they will only consider the most likely path from 2 under input *a*: the path that takes the system from State 2 directly to State 0. Once the system is in State 0, if it receives any input, it will necessarily transition to State 2; they will thus estimate the probability of the final state being State 0 as zero. The mathematical form of the heuristic is shown in Table 2.

**Table 2.** Alternative neglect (AN), a heuristic that deviates from the normative standard of Table 1. Here, *ϵ*(1*_a_*) is equal to argmax*_k_ P*(*k*|1*_a_*), the most likely next state if one starts in 1 and takes action *a*; replacing the summation over different alternatives by the *ϵ* term is the key simplification of the AN heuristic, that constructs the relevant path in a “greedy” fashion.

Task	Hidden Form with Alternative Neglect
Prediction	argmax*_i_*(*P*(*i*|*ϵ*(1*_a_*)*_b_*)*P*(*ϵ*(1*_a_*)|1*_a_*))
Explanation	*P*(3|*ϵ*(1*_b_*)*_b_*)*P*(*ϵ*(1*_b_*)|1*_b_*) < *P*(3|*ϵ*(1*_a_*)*_a_*)*P*(*ϵ*(1*_a_*)|1*_a_*)
Control	argmax_{*i*,*j*}_*P*(3|*ϵ*(1*_i_*)*_j_*)*P*(*ϵ*(1*_i_*)|1*_i_*)

## EXPERIMENTS 1 AND 2: THE EFFECT OF FSM COMPLEXITY AND HIDDEN INFORMATION

In Experiments 1 and 2, participants engaged with a simple chatbot, defined by a finite-state machine, and after a free exploration phase, we measured their ability to predict, explain, and control this simulated partner. We manipulated the complexity of FSMs by introducing higher (*hard* FSM) or lower (*easy* FSM) number of probabilistic transitions. We also varied uncertainty by hiding some of the information relevant for decision-making.

### Method: Experiment 1

#### Participants.

We recruited 97 participants from the U.S. (49 men and 48 women; ages 18–47; *M*_*age*_ = 27.34, *SD*_*age*_ = 6.73) online via Prolific and paid them $2 with a bonus of up to $2 based on their performance. The participants had normal or corrected-to-normal vision and completed the study on a desktop or laptop computer in about ten minutes. Informed consent was obtained from all participants via an electronic form. The study protocol was approved by the Carnegie Mellon University Institutional Review Board.

#### Materials.

We created a Chatbot Interaction Task in which participants communicated with a “chatbot” using a fixed set of emoji icons and then answered questions that measured their ability to predict, explain, or control the chatbot’s behavior.

The chatbot followed a set of rules defined by a finite-state machine with four states (chatbot’s messages) and two inputs (participant’s responses) that determined the transitions between them. Some state-input combinations led to the same next state every time, *i.e.*, were *deterministic*. Others could lead to multiple next states with a certain probability, *i.e.*, were *probabilistic*. There were also *predetermined* transitions, where the next state did not depend on the input.

Participants were randomly assigned to one of two finite-state machines (see Figure 1) that varied in the number of probabilistic and deterministic transitions. The *easy* machine had two probabilistic and six deterministic transitions, including two predetermined ones. The *hard* machine had five probabilistic and three deterministic transitions, including one predetermined one.

#### Procedure.

The procedure consisted of a learning phase (45 interactions), followed by a test phase (20 questions), and a short questionnaire.

**Figure 2.**
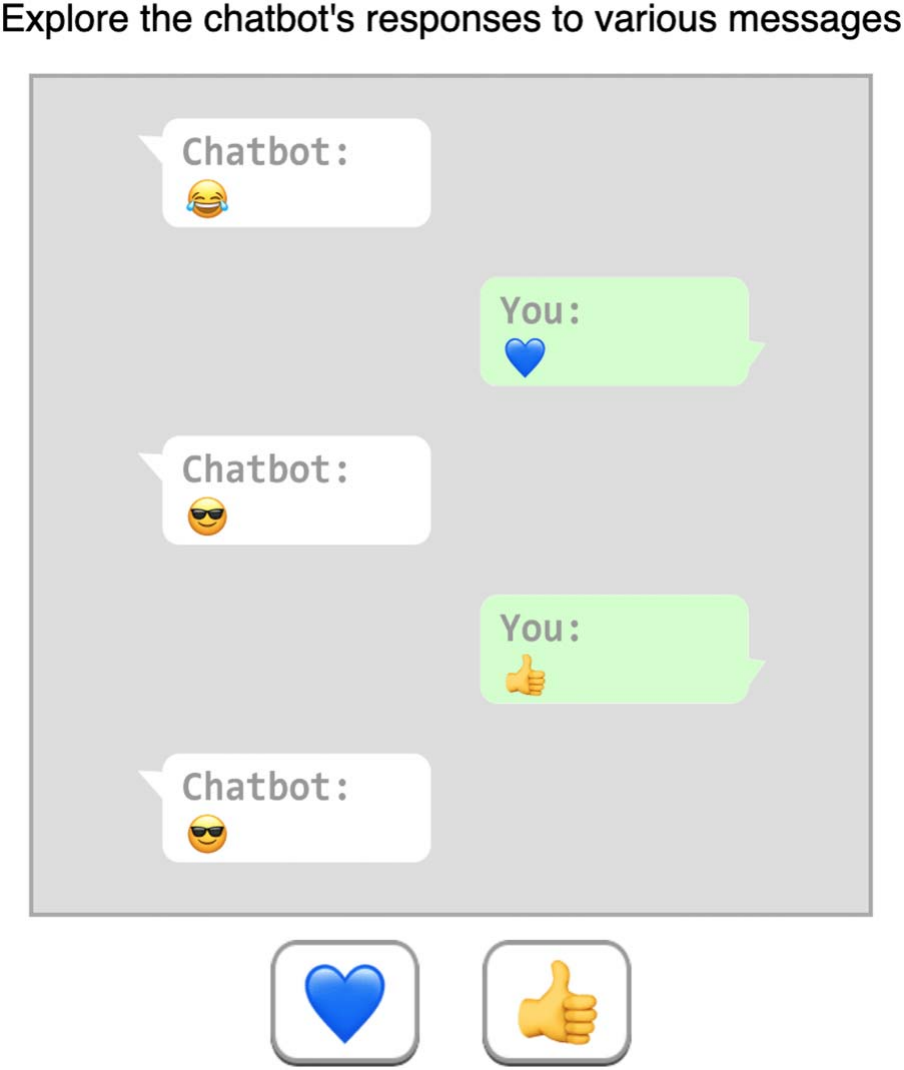
**Interaction with the chatbot during the “free exploration” learning phase**. The conversation initiated with a random emoji sent by the chatbot. Participants subsequently selected one of two response options using on-screen buttons. Following their choice, the chatbot responded based on the structure of the finite-state machine.

At the beginning of the *learning phase*, participants were told that the chatbot’s responses follow a certain pattern and were asked to freely interact with the chatbot “to get a sense of how it responds to different messages so that they would be able to explain, predict, and control its behavior.” They were also asked not to use any outside resources or assistance. The chatbot begins in a random state (see Figure 1), emitting the associated emoji. Participants respond by choosing one of the two emoji icons (corresponding to *a*, or *b*) and instantly get the next reaction of the chatbot, which depends on their input and the previous message from the chatbot (Figure 2).

**Figure 3.**
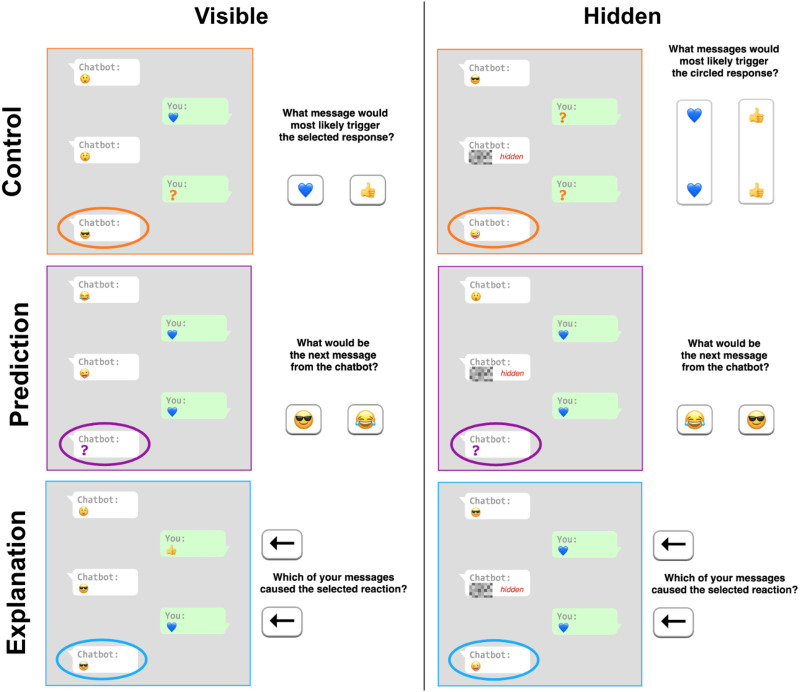
**Examples of questions presented during the test phase**. Participants selected their answers using the buttons on the right side of the screen.

In the *test phase*, participants were randomly assigned to one of three test conditions that assessed their ability to predict, explain, or control the chatbot’s behavior. All test tasks were presented as episodes of conversation with the chatbot, exactly paralleling the format of Table 1, with a two-alternative forced-choice question corresponding to the test condition. As a within-subjects variable, a form of test question (*visible* or *hidden*) was manipulated by presenting or hiding an intermediate message from the chatbot (see Figure 3). A total of 20 questions—ten hidden and ten visible—were asked in random order. Participants received 10 cents for each correct answer as a bonus payment.

The goal of the **prediction** task was to see if participants could correctly anticipate the chatbot’s next response by looking at past interactions. Visible form included a State_1_–Input_1_–State_2_–Input_2_–[Question] combination along with a question (*What would be the next message from the chatbot?*) and two states as answer options. The hidden form was identical except for State_2_ being concealed.

Visible **control** tasks were presented as State_1_–Input_1_–State_2_–[Question]–State_3_ episodes with a question (*What message(s) would most likely trigger the selected response?*) and two inputs (*a* or *b*) as answer options. Hidden control tasks contained only State_1_ and State_3_ with State_2_ and both inputs being hidden. Answer options included two (out of four possible) combinations of Input_1_ and Input_2_. Participants had to determine which message or combination of messages would evoke a specific response from the chatbot.

In the **explanation** condition, the task was to decide which of the previous messages caused the chatbot’s final reaction. In the instructions, we emphasize that the message that causes the final response need not be the one that occurred immediately before: “It can sometimes be the case, for example, that once a certain action is taken, the next action has little or no ability to change the outcome. In this case, the earlier action may have been the cause.” Conversation episodes were presented as State_1_–Input_1_–State_2_–Input_2_–State_3_ with State_2_ being visible or hidden along with a question (*Which of your messages caused the selected reaction?*) and two answer options—buttons pointing at Input_1_ or Input_2_.

### Method: Experiment 2

Post-experiment feedback in Experiment 1 suggested that some participants (10%) did not believe that the test questions had correct or incorrect answers at all, and more than half (57%) estimated their accuracy at a chance level. We assumed that the free exploration format with no feedback or a clear goal at the learning phase may have contributed to a decrease in motivation to study the pattern. In order to address this issue, we slightly changed the learning phase by introducing “preview questions”: examples of the kinds of questions that would be asked in the test phase followed by immediate feedback on the accuracy.

#### Participants.

We recruited 266 participants (134 men and 132 women) with an age range of 18–47 years (*M*_*age*_ = 28.54, *SD* = 7.34) using the same sampling strategy as in Experiment 1.

#### Materials.

The task was identical to Experiment 1.

#### Procedure.

Experiment 2 followed a similar procedure to Experiment 1, with the addition of five preview questions in the learning phase that corresponded to the participant’s test condition (prediction, explanation, or control). The preview questions were intended to motivate participants to learn from their interactions with the system and to show that the task had correct and incorrect answers. We presented the preview questions after the 10th, 15th, 18th, 25th, and 30th trials. We only used preview questions for interactions that had a normatively correct answer. All preview quesitions were presented in visible format (see Figure 3).

### Results

Our results are twofold. Concerning our Hypothesis 1, we find, as expected, that while there are differences in performance between prediction, explanation, and control tasks under conditions of full information, the performance across these tasks equalizes when information is hidden.

Concerning Hypothesis 2, we find strong evidence that, under hidden information, people use the alternative neglect heuristic in preference to the fully Bayesian-normative answer, across all three tasks.

We present our results in detail below.

#### Comparison of Accuracy Between Experiments 1 and 2.

We first evaluated the impact of previewing and receiving feedback on five test questions during the learning phase by comparing the average accuracy between Experiment 1 (*M* = 0.60, *SD* = 0.15) and Experiment 2 (*M* = 0.61, *SD* = 0.14) across three test conditions using *t*-tests for independent samples (see Table 3). No statistically significant differences were found. Given that modifications in the learning phase did not influence performance, we decided to combine the data from both experiments in our subsequent analyses to enhance the statistical power of our results. The combined sample consisted of 363 participants (180 women and 183 men; *M*_*age*_ = 28.22, *SD*_*age*_ = 7.20).

**Table 3.** Comparison of FSM accuracy between Experiments 1 and 2 across different test conditions.

Test condition	Experiment 1		Experiment 2		*t* (119)	*p*
	*M*	*SD*	*M*	*SD*		
Prediction	.62	.19	.59	.14	0.73	.468
Explanation	.56	.11	.56	.12	0.20	.844
Control	.64	.12	.69	.13	−1.54	.125

*Note*. Means and standard deviations were calculated from aggregated participant data.

#### Mental Model Accuracy.

**Figure 4.**
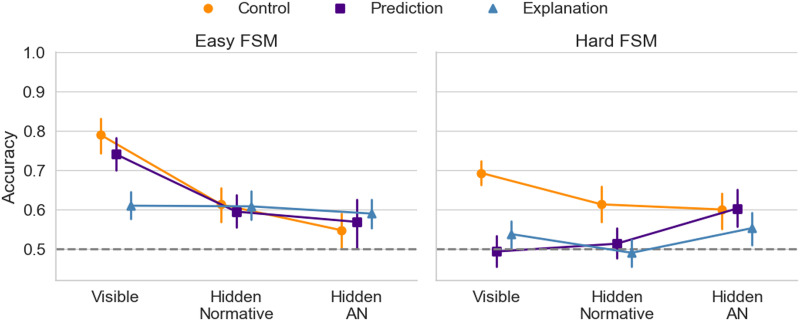
**Experiments 1 and 2: Accuracy based on participants’ mental models**. Means and 95% CIs were calculated from aggregated participant data (*N* = 363).

Following the discussion in the Mental Models and Dynamical Systems section, we present the mental model accuracy, based on the probabilities that individual participants observed during the learning phase.^1^ Figure 4 shows that participants were able to predict, control, and explain both visible and hidden forms of questions in the *easy* FSM condition. However, in the *hard* FSM condition, only control remained at the above chance level across all forms of questions.

We conducted pairwise *t*-tests with Benjamini-Hochberg adjustment for multiple comparisons to examine the differences between test conditions. In the *easy* FSM condition, participants performed worse in explanation (*M* = .61, *SD* = .14) when responding to the visible form of questions compared to prediction (*M* = .74, *SD* = .17, *t*(118) = −4.57, *p* < .001) and control (*M* = .79, *SD* = .18, *t*(114.09) = −6.14, *p* < .001). No other statistically significant differences were found in the *easy* FSM.

In the *hard* FSM condition, there was a performance advantage in control (*M* = .69, *SD* = .12) compared to prediction (*M* = .49, *SD* = .16, *t*(111.46) = 7.77, *p* < .001) and explanation (*M* = 0.54, *SD* = 0.13, *t*(118.37) = 6.78, *p* < .001) for the visible form of questions. Similar effects were observed in the hidden condition with accuracy in control tasks (*M* = .61, *SD* = .18) being higher than in prediction (*M* = .51, *SD* = .16, *t*(116.9) = 3.30, *p* = .002) and explanation (*M* = .49, *SD* = .14, *t*(112.80) = 4.24, *p* < .001) task.

Overall, the results from the mental model accuracy comparison revealed that both FSM type (easy/hard) and question format (visible/hidden) influence participants’ performance in prediction, explanation, and control. However, this influence is not consistent and varies depending on the task. This variability can be attributed to the differing complexities of the questions across tasks. Consequently, we extended the analysis to consider both the accuracy of the answers and the complexity of each question.

#### Bayesian Evidence (*R*_*i*_) Analysis.

**Figure 5.**
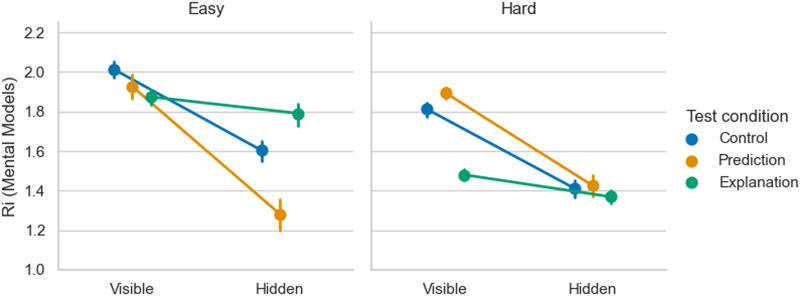
**Experiments 1 and 2: Mean values of *R*_*i*_ (strength of Bayesian evidence for a given test question) and 95% confidence intervals**. Calculations were made using aggregated participant data. *R*_*i*_ = 0 corresponds to equally probable, that is, uninformative, choice options in a test task. *R*_*i*_ = 2.4 corresponds to the easiest cases where one option has a probability of 1 given the participant’s mental model, and the other option equals zero.

To account for the fact that test questions may vary in difficulty depending on the magnitude of answer option probabilities, we calculated *R*_*i*_ values that represent the strength of Bayesian evidence for a given test question *i*. Larger *R*_*i*_ values correspond to questions that should be easier to answer, assuming that participants interact with their mental models in a Bayesian fashion. *R*_*i*_ equals zero when the options are indistinguishable and, therefore, uninformative of the participant’s mental model. As shown in Figure 5, there are differences across conditions in both mean *R*_*i*_ values and their variability from question to question. Put simply, depending on task, machine, and condition, some questions are harder than others. A direct comparison of accuracy (how many times the higher-probability answer was chosen) would neglect this fact, and potentially obscure the extent to which participants were able to make use of the available information. Measuring the *R*_*i*_ values, however, allows us to correct for this effect.

**Figure 6.**
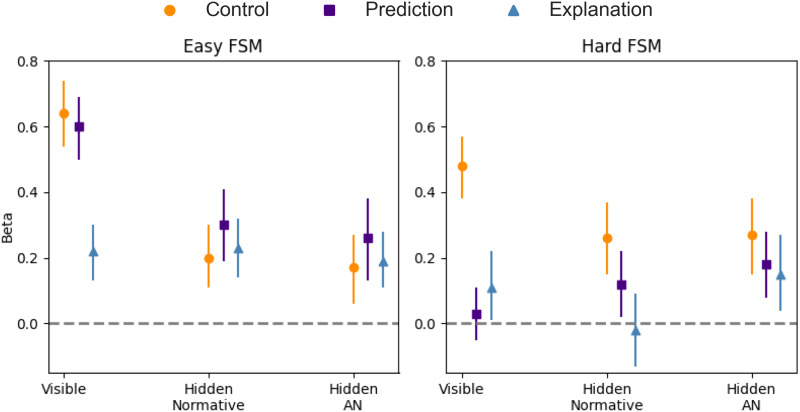
**Experiments 1 and 2: Beta values and 95% confidence intervals from logistic regression models with *R*_*i*_ predicting accuracy**. In the Hidden Alternative Neglect (AN) case, accuracy was calculated based on the alternative neglect assumption.

In particular, we conducted a logistic regression analysis, using the Bayesian log-evidence of answer choices (*R*_*i*_) as a predictor of accuracy (see Figure 6). In the *easy* FSM condition, the answer choice evidence (*R*_*i*_) was a statistically significant predictor of accuracy across all conditions, suggesting that participants generally used evidence in a Bayesian manner. Mirroring the mental model accuracy results, Figure 6 shows that *R*_*i*_ was a better predictor of correct responses for prediction and control tasks compared to the explanation task.

Importantly, when the intermediate state was hidden, many of the differences between prediction, explanation, and control disappeared, while beta values for *R*_*i*_ remained significantly greater than zero. In the *hard* FSM condition, and assuming the normative use of models according to Table 1, *R*_*i*_ remained a positive, statistically significant predictor of accuracy across visible and hidden forms of questions, but only for control. Explanation was slightly above the chance level in the visible form, and prediction was slightly above the chance level in the hidden (normative) form of questions. When faced with the hidden condition, in other words, participants did not reliably make use of information in a proper Bayesian fashion.

However, and importantly, the beta values for *R*_*i*_ became similar (and significantly above chance level) across all three tasks and both machine complexities, when we use the alternative neglect assumption. This suggests that people were indeed able to make use of information contained in their mental models, but only by way of computationally-simplifying heuristics that avoided detailed consideration of alternative, less likely, possibilities.

To test this hypothesis, we compared the predictions for behavior of the alternative neglect model (Table 2) to the normative model, using a likelihood-ratio test. In particular, the two models (alternative neglect and normative) provide a probabilistic prediction for how participants answered each question in the test suite; in the simplest case, the two models have one free parameter, *β*, the sensitivity to evidence. This enables us to compute the maximum log-likelihood of each model, given the participant-level data on how each question was answered. The alternative neglect model provided a better fit for prediction (Δ Log-Likelihood (*L*) = 173.88, *p* < .001) and control (Δ*L* = 12.72, *p* < .001) than the normative model, with the explanation task (slightly) favoring the normative model (Δ*L* = 4.00, *p* < .047). In the hard FSM condition, the alternative neglect model significantly outperformed the normative model across all three tasks, including explanation (Δ*L* = 17.81, *p* < .001), prediction (Δ*L* = 136.02, *p* < .001), and control (Δ*L* = 55.22, *p* < .001).

#### Recency Effects in Explanation.

As an attempt to explore possible reasons for the differences obtained in the explanation task compared to prediction and control, we checked whether participants were biased toward choosing the most recent input. As expected, participants were slightly more likely to choose the most recent response when making causal judgments (*M* = 0.58, *SD* = .18), which was statistically significantly different from 50% (*t*(120) = 4.7, *p* < 0.001).

### Discussion: Experiments 1 and 2

The primary goal of Experiments 1 and 2 was to investigate how individuals predict, explain, and control dynamical systems under varying levels of complexity and uncertainty. We assumed that limiting the availability of information would force participants to rely on their—presumably probabilistic—mental models instead of using more simple memorization and pattern-matching strategies.

When full information about the system was available, performance significantly varied across prediction, explanation, and control tasks. Specifically, in the *easy* FSM condition, participants performed worse in the explanation task compared to the prediction and control tasks. This finding aligns with Pearl’s (2009) suggestion that counterfactual causal judgments, which are required in the explanation task, are more challenging. Counterfactual reasoning involves suppressing factual information and imagining alternative scenarios, making it more cognitively demanding than the hypothetical (factual) reasoning involved in control and prediction tasks.

At the same time, *hard* FSM condition revealed that control was performed with higher accuracy than prediction and explanation, suggesting that control might be more robust to the higher stochasticity and complexity of the environment. Notably, performance in a normatively identical prediction task was at chance level, indicating that participants may have relied on simple rules of thumb or recollection of instances (similarly to Brehmer & Elg, 2005; Gonzalez et al., 2003) rather than probabilistic mental models of the dynamical system. On the surface, this seems to conflict with Pearl’s hierarchy that understand control as coming online only once prediction is available. However, some caution might be warranted, because our free exploration learning phase, where participants made interventions by selecting inputs of their choice, might have shared more similarities with the control than the explanation or prediction tasks. This similarity in task demands could have facilitated the learning of model-free strategies for maintaining control, contributing to superior performance in this task without benefiting prediction and explanation.

Interestingly, the introduction of hidden information, combined with the alternative neglect heuristic, equalized the performance differences observed across prediction, explanation, and control tasks. This equalization was observed in both the *easy* and *hard* FSM conditions. According to our theoretical approach, when people rely on probabilistic mental models, they ought to produce similar performance across all three tasks—the rules in both Table 1 and 2 do not vary in algorithmic complexity between the three tasks—and this is precisely what we find in the case of hidden information.

Overall, findings in Experiments 1 and 2 suggest that people use distinct and different cognitive strategies to predict, explain, and control dynamical systems when dealing with visible and hidden information, and suggest that people switch to more general strategies in the presence of the unknown. This might occur if individuals rely on simple familiarity-based strategies or state-input-state associations in the visible cases, but shift, when information is only partial, to a more generalized set of abilities that draw on probabilistic representations.

Unfortunately, the between-subjects design employed in Experiments 1 and 2, while offering several advantages, has intrinsic limitations. Most notably, each participant was exposed to only one task—either prediction, explanation, or control,—and as a result we could not evaluate the relationship between these abilities within the same person. To address this limitation, we designed Experiment 3, where we adopted a within-subjects design, enabling participants to engage in all three tasks.

## EXPERIMENT 3: RELATIONSHIP BETWEEN INDIVIDUAL PERFORMANCE IN PREDICTION, EXPLANATION, AND CONTROL TASKS

In Experiment 3, we examined the potential shared mental model underlying abilities to predict, explain, and control a dynamical system. Participants had an extended learning phase and then performed three test tasks in a counterbalanced order. We had to reduce the number of question in the test tasks to mitigate the possible effects of order and fatigue.

### Method

#### Participants.

The same sampling strategy as in Experiments 1 and 2 was used to recruit 240 participants (120 men and 120 women; age range 18–47; *M*_*age*_ = 28.89, *SD*_*age*_ = 7.07) via Prolific.

#### Materials.

The task remained identical to that used in Experiments 1 and 2.

#### Procedure.

The *learning phase* was as in Experiment 1, but the number of learning trials was increased from 45 to 60. Preview questions (see Experiment 2) were not used, as they did not seem to affect the results.

In the *test phase*, a within-subjects design was used, meaning each participant performed all three test tasks (prediction, explanation, and control). However, in each of the test conditions, there were only 10 test questions, with the first five in visible format, and the last five in hidden format. The order of test tasks was counterbalanced. Since the number of trials was halved compared to previous experiments, twice as many participants were recruited to achieve similar statistical power as in Experiments 1 and 2.

### Results and Discussion

Concerning Hypothesis 3, our main result for Experiment 3 is inconclusive: we found some correlations in a participant’s performance on different tasks, but whether or not they were present varied with both machine complexity and task pair.

#### Accuracy in Experiment 3 vs. Experiments 1 and 2.

Although Experiment 3 had a smaller number of test questions in each test condition, we expected that an extended learning phase and a larger sample size would result in accuracy levels and statistical power similar to those in Experiments 1 and 2. Table 4 shows that accuracy in Experiment 3 was similar to Experiments 1 and 2.

**Table 4.** Comparison of FSM accuracy between Experiment 3 and Experiments 1 and 2 (combined) across different test conditions.

Test condition	Experiments 1 and 2		Experiment 3		*t* (359)	*p*
	*M*	*SD*	*M*	*SD*		
Prediction	0.60	0.15	0.61	0.17	−0.80	.423
Explanation	0.56	0.12	0.55	0.16	0.85	.397
Control	0.67	0.13	0.66	0.16	0.79	.431

*Note*. Means and standard deviations were calculated from aggregated participant data.

#### Recency Effects in the Explanation Task.

In the explanation task, participants selected the most recent input as the cause in 64% (*SD* = .16) of cases, which is statistically significantly different from 50% (*t*(239) = 13.22, *p* < .001). Thus, similarly to Experiments 1 and 2, there was a bias toward the most recent event when making causal judgments.

#### Relationship Between Prediction, Explanation, and Control.

We hypothesized that if participants rely on a common mental model to answer prediction, explanation, and control test questions, there would be a correlation between these abilities. To test this idea, we used the approach described in the Mental Models and Dynamical Systems section to find beta values that best describe an individual’s responses, that is, maximize the sum of log likelihoods across all trials in a given experimental condition. Beta values greater than zero suggest that participant’s responses are in line with the probabilities of state-input-state transitions observed in the learning phase.

**Figure 7.**
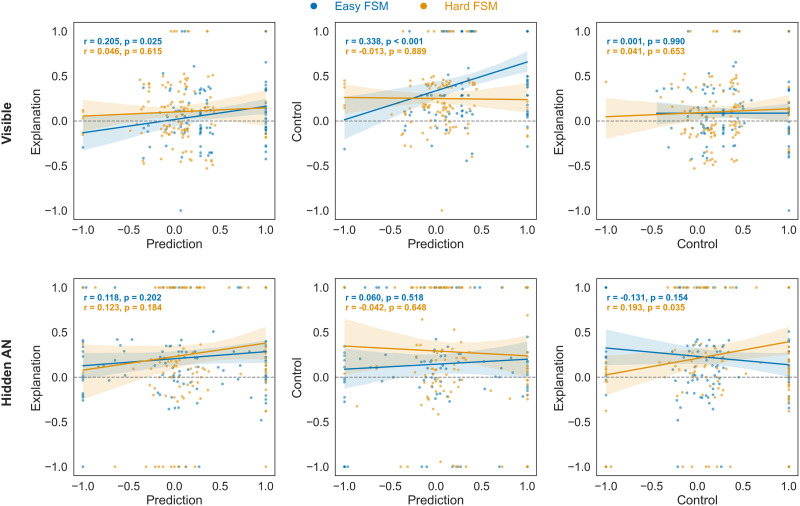
**Experiment 3: Correlations between individual-level beta values** for prediction, explanation, and control tasks in questions with *visible* (tow row) and *hidden* (bottom row) intermediate states.

Indeed, in the *easy* FSM condition, we found evidence of some relationships between performance in the tasks. Figure 7 shows that there were weak positive associations between participants’ betas in prediction and control tasks (both in visible and hidden forms of questions), and a weak correlation between explanation and prediction tasks in the visible form of questions. Overall, there is some evidence that task performance is driven by the quality of an underlying, generalizable mental model when it comes to prediction and control questions in the *easy* FSM condition, but across both machines and all tasks there is a great deal of noise.

There are two main sources of noise. Firstly, in contrast to Experiments 1 and 2, Experiment 3 requires use to measure a participant’s underlying performance. In order to avoid participant fatigue, we had a smaller number of test questions for each task (five questions for each combination of visible/hidden and prediction/explanation/control). This leads to noise in estimating a participant’s underlying abilities. Secondly, this noise is exacerbated by the fact that the tasks are all quite difficult, and many participants are operating at chance. While this averages out at the population level, at the individual level it serves to obscure the more delicate effects associated with the quality of the mental model.

Imagine, as a thought experiment, two types of participants. One has a mental model that enables them to achieve 70% accuracy on (say) both prediction and control, near the upper-level of our observed performance. The other has no mental model at all, and is forced to guess at random. Even in this case, where possession of a mental model is the sole driver of all performance differences, and that possession is an all-or-nothing effect, noise effects at the test stage from the finite number of questions mean that we should expect a correlation between the *β*s of the two tasks of only approximately 0.17, and we would have a false-negative (Type II error) rate, for *N* = 200, of approximately 20%. One possibility is to increase the number of questions; if we were to go from five questions to ten questions, for example, we could expect a correlation of approximately 0.29, with negligible Type II error. However, we were concerned about participant fatigue; as we increase the number of questions, we expect accuracies to go down.

Another possibility is to raise the performance of those who do acquire the model: in our simplified thought experiment, if those who acquired a mental model were to rise from 70% accuracy to 80%, the increased variability means that correlations would rise to approximately 0.35, with negligible Type II error. As we shall see, Experiment 4, below, which looks at the question of cross-training, has this side-effect.

## EXPERIMENT 4: EVIDENCE FOR CROSS-TRAINING ABILITIES

Experiment 4 aimed to establish causal relationships between the abilities to predict, explain, and control dynamical systems. This was achieved by introducing a reinforcement learning format focused on one of these tasks, followed by a transfer phase involving the other two tasks. To ensure subjects actually learned the training task, we set an accuracy threshold of 65% (a chance level was 50%) as a prerequisite to advance to the subsequent phase. Participants who did not meet this threshold were excluded from the transfer test phases and subsequent analyses.

We hypothesized first that, to the extent that mental models are in play, they should enable the transfer of knowledge from one task to another. Model-free heuristics and simpler strategies, by contrast, would be less effective when the context shifted. Experiment 4 probes the extent to which training on one task leads to the creation of generalizable mental models.

Our second hypothesis, which follows the same logic as that for Experiment 3, was that, to the extent that mental models are in play, we should expect correlations in performance at the participant level: those who learned the task better should also perform better on the other tasks. While we did not see the parallel effect in Experiment 3, we considered this worth investigating a second paradigm. The analysis of the previous section suggested that low performance was a limitation on statistical power in Experiment 3; a side-benefit of training provided in Experiment 4 might be to raise the ceiling on performance, potentially increasing the variability between subjects and making differences easier to detect.

### Method

#### Participants.

A total of 210 participants were recruited via Prolific (106 men and 104 women; age range 19–47, *M*_*age*_ = 32.5, *SD*_*age*_ = 7.4). Twenty five additional participants did not reach the pre-defined 65% accuracy threshold after three attempts in both visible and hidden blocks of questions and were subsequently excluded from further analysis^2^.

The study took approximately 18 minutes. Participants were paid $3 with a bonus up to $5.5 based on their performance: $1.5 for reaching the accuracy threshold in the feedback learning phase and $0.10 for each correct answer in the subsequent transfer test phases.

#### Materials.

The experimental task was identical to that used in Experiments 1–3.

#### Procedure.

Experiment 4 comprises three phases: the free interaction phase, feedback learning phase, and transfer test phase.

The *free interaction phase* is similar to the learning phase in Experiments 1–3 but has only 16 trials. The goal is to familiarize participants with the dynamic interaction task without giving them enough trials to fully grasp the finite-state machine.

In the *feedback learning phase*, participants respond to prediction, explanation, or control test questions and receive feedback on their accuracy after each trial. This phase is divided into two parts: (1) visible and (2) hidden. Participants are informed that they must achieve a 65% accuracy threshold in at least one of these parts to get admitted to the next part of the study. Firstly, participants respond to 15 questions in the *visible* format. Those who fail to meet the accuracy threshold are given additional attempts to until they either achieve the threshold or complete all three attempts. Subsequently, they are presented with 15, 30, or 45 questions in the *hidden* format, adhering to the same procedure.

The *transfer test phase* consists of two types of test tasks (20 trials each, 10 visible and 10 hidden) that were not presented earlier. For instance, some participants learned to predict through a feedback learning format and were subsequently tested–without feedback–on their abilities to control and explain. Conversely, others learned to explain and were tested on their abilities to control and predict, and so on. The order of transfer test phases was counterbalanced.

### Results and Discussion

#### Feedback Learning Phase.

The vast majority of participants (70%, *n* = 146) passed the threshold in both visible and hidden trials; 39 participants passed the threshold in the visible condition but not in the hidden, and 25 did the opposite. The lowest accuracy was observed in the explanation condition with the *hard* FSM (62% for visible, 65% for hidden form of question), while the highest accuracy (82%) was in the visible control condition with the *easy* FSM^3^.

The mean number of attempts in the feedback learning phase was 1.81 (*SD* = 0.85) out of a maximum of three. Consistent with our expectations, the more challenging experimental conditions required participants to make more attempts to achieve the 65% accuracy threshold. Specifically, participants interacting with the *hard* FSM required more attempts (*M* = 1.93, *SD* = 0.84) compared to those with the *easy* FSM (*M* = 1.71, *SD* = 0.86); *t*(418) = 2.71, *p* = .007. Similarly, participants in the explanation condition also made more attempts (*M* = 1.99, *SD* = 0.86) than those in the control and prediction conditions combined (*M* = 1.74, *SD* = 0.84); *t*(418) = 2.82, *p* = .005. Lastly, when comparing the number of attempts between the *hidden* and *visible* forms of questions, participants required more attempts in the hidden form (*M* = 1.98, *SD* = 0.86) than in the visible form (*M* = 1.65, *SD* = 0.82), *t*(209) = 3.99, *p* < .001.

#### The Effect of Training Conditions on Participants’ Beta Values.

Similar to Experiment 3, for each participant, we determined beta values using the methodology described in the section Mental Models and Dynamical Systems. Beta values for the main task (learning via feedback) were determined based on the final attempt. Values above zero indicate behavior consistent with the probabilistic structure of the finite-state machine.

**Figure 8.**
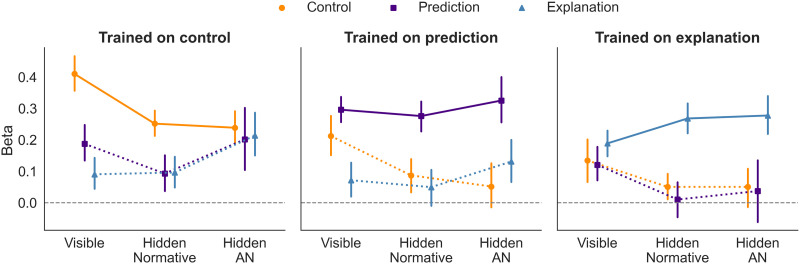
**Experiment 4: The effect of training conditions on participants’ beta values**. Means and 95% confidence intervals are displayed. Solid lines represent performance during the final attempt of the feedback learning phase, while dotted lines denote transfer test phases. Beta values above zero indicate that responses are consistent with the underlying finite-state machine.

Figure 8 illustrates the influence of training conditions on participants’ beta values. Training participants on control demonstrated a significant transfer to prediction and explanation skills both in situations with visible and hidden information. Training focused on prediction showed discernible transfer to the control task and a possible transfer to the explanation task. However, training on explanation displayed limited transferability. Participants exhibited transfer only in the scenario with the visible intermediate state and struggled to apply their mental model in the hidden context.

In summary, mastery in control equips participants with the skills for prediction and explanation. While proficiency in prediction aids in control, expertise in explanation appears to offer limited transfer benefits in the hidden form of questions. Overall, our results suggest that interventions (i.e., control actions) are essential for constructing a flexible mental model of a dynamic system.

#### Relationship Between Prediction, Explanation, and Control.

Introducing a 65% accuracy threshold in the feedback learning phase ensured that participants achieved mastery in either controlling, predicting, or explaining the dynamic system depending on their experimental group. In this context, the absence of a correlation between abilities cannot be simply attributed to the the inability to learn the dynamic system at all.

**Figure 9.**
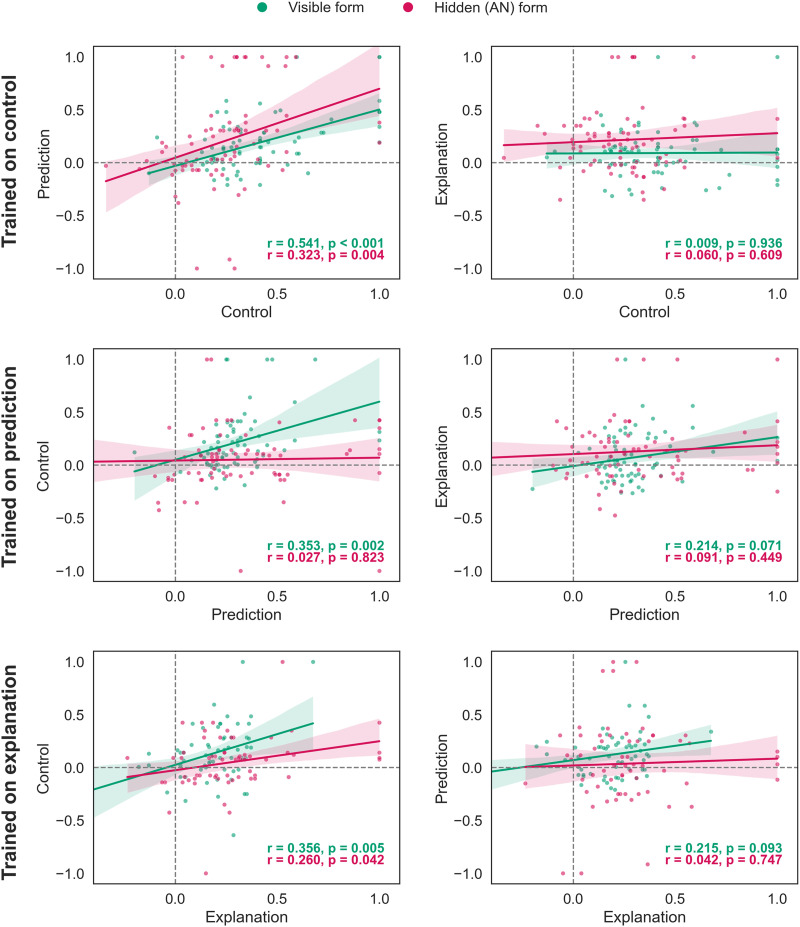
**Experiment 4: Relationships between participant beta values in learned via feedback (*x*-axis) and no-feedback transfer (*y*-axis) tasks**. Values above zero indicate that responses correspond to the finite-state machine.

Figure 9 illustrates the relationships between participant performance in tasks learned via feedback and those in the no-feedback transfer test phase. The correlation plots reveal several patterns. First, proficiency in control tasks positively correlates with prediction skills and vice versa. Second, there were no statistically significant correlations between explanation in its hidden form. However, there was a statistically significant positive relationship between performance in the visible explanation and control conditions when trained on explanation. Notably, this relationship was not observed in the reverse case (training on control).

In summary, while there is evidence that control and prediction are closely linked, explanation appears to be a rather distinct ability. It might be based on a different cognitive foundation or involve supplementary processes, such as counterfactual reasoning.

## DISCUSSION AND CONCLUSION

The goal of this study was to investigate how individuals predict, explain, and control dynamical systems under varying levels of complexity and uncertainty. We manipulated the availability of information about the systems (visible or hidden forms of questions) and the complexity of the systems (easy or hard FSM), in order to better understand the relationship between the three tasks, and the extent to which abilities might be understood to rely on a common representational source.

To do this, we introduced a novel methodology for examining human performance: a dynamic interaction task involving a chatbot. We operationalized prediction, explanation, and control in a computationally compatible manner and developed strategies to ensure comparability of performance across these tasks.

Instead of relying on the actual finite-state machines, in Experiments 1–3, we utilized the observed probabilities of transitions to infer participants’ mental models. By doing so, we eliminated distortions that could have arisen from unpredictable response choices during the free exploration learning phase. We also accounted for differences in test questions by employing a Bayesian evidence metric (*R*_*i*_) that considers the discriminability of answer options, and addresses variations in question informativeness with respect to participants’ mental models. These measures enabled us to compare human performance across seemingly incomparable tasks and gain deeper insights into the underlying cognitive mechanisms involved in prediction, explanation, and control within dynamical environments.

Our paradigm enables us to investigate all three tasks on an equal footing. It does have limitations, however; one limitation is the difficulty of giving participants enough time to learn the system, and testing them across six different modalities (three different tasks, and a visible/hidden condition), without leading to fatigue. In Experiment 3, in particular, our decisions to limit the total number of test questions led to limitations on statistical power at the individual level. Despite these limitations, however, our work provides three major findings.

The first major finding of our work, replicated in both Experiment 1 and 2, and Experiment 3, is that in the presence of full information, participants performed best on control, but that things change dramatically in the presence of hidden information: prediction, explanation, and control performance equalizes when information is hidden.

The second major finding is that people rely on an alternative neglect heuristic when information is hidden and they need to reason through multiple possibilities. In such situations, our results suggest, people tend to focus on the most likely alternative instead of considering all possible scenarios. This second finding complements earlier work on the alternative neglect phenomena (Fernbach et al., 2010, 2011), demonstrating that complex stochastic environments can trigger the reliance on alternative neglect even in backward-reasoning, explanatory, judgments. Our study goes beyond classical alternative neglect experiments, which used description-based tasks because we adopted an experience-based approach, where participants actively engaged with and learned from the dynamical systems. This distinction is important because research on the description-experience gap has shown that judgments in verbal tasks may not always align with mathematically identical judgments made from personal experiences (Hertwig & Erev, 2009). By employing an experience-based paradigm, our study provides insights into decision-making processes in realistic contexts and sheds light on the use of heuristics in complex and uncertain environments.

While reliance on the alternative neglect heuristic is, formally, non-Bayesian and thus (for example) exploitably non-optimal, it does not necessarily indicate sub-optimal behavior. Summing the probabilities over all possible scenarios may not be feasible under cognitive constraints, and the maximum-likelihood alternative neglect heuristic may well be more robust to computational errors than an attempt to reproduce the normative demand. This is a familiar result in a new domain: simple heuristics often outperform more complex, normative strategies in ecologically valid high-uncertainty tasks, as demonstrated earlier (Brighton & Gigerenzer, 2012; Neth & Gigerenzer, 2015).

The third major finding of our work concerns the phenomenon of cross-training. Our results suggest that cross training is possible—an important prediction of theories that talk in terms of mental models—but that there are asymmetries in how it works. In particular, training on control appears to lead to abilities in prediction and explanation, but these effects are much weaker under training for prediction and explanation. We find this both at the population level, and (for the case of cross-training between prediction and control) at the within-subjects level.

In the mental model framework, this suggests that teaching a person to control a system is the most effective way to give people domain-general mental models that then enable them to predict and explain. Conversely, teaching people to explain, or predict, leads to more specific abilities.

These training results complicate a simple story where abilities are always emerge simultaneously from a common root. Consider, for example, Figure 9; the population trained to control has similar explanation abilities as the population trained to explain. However, the population trained to explain does not have the prediction abilities of the control group. Simply possessing one skill does not necessarily imply the other; it appears to matter how the skill was acquired, a phenomenon that also appears at the individual, within-subject, level as well.

All of our results come from an interaction task that is conceptually clean, but also somewhat distant from ordinary experience—even the most laconic communicator has more than two emojis and, in contrast to our design, brings a great deal of domain-specific priors to the task. A major open question for this kind of work is how to extend our paradigm to more ecologically-rational situations, to see how these fundamental results interact with the context-rich environments that actually obtain in real-world interactions. Leveraging recent progress in LLMs like GPT-4 (OpenAI, 2023, Trott et al., 2023), one might imagine, for example, a natural-language version of our task, where people interact with a more realistic companion by choosing different messages to send to achieve a desired outcome, predicting more or less likely responses, or explaining why a conversation took the direction it did.

## ACKNOWLEDGMENTS

We thank Victor Odouard, Victor Møller Poulsen, Sarah Marzen, Arseny Moskvichev, Marina Dubova, Keiji Ota, and an anonymous referee for helpful conversations and comments. This work was supported in part by the Survival and Flourishing Fund.

## DATA AVAILABILITY STATEMENT

Data, analysis scripts, and materials for data collection can be found on the Open Science Framework website: https://osf.io/d8agn/.

## COMPETING INTEREST STATEMENT

The authors declare no competing interests.

## Funding Information

This work was supported in part by the Survival and Flourishing Fund.

## Author Contributions

Roman Tikhonov: Conceptualization; Formal analysis; Investigation; Methodology; Visualization; Writing–Original draft; Writing–Review & editing. Simon DeDeo: Conceptualization; Formal analysis; Funding acquisition; Investigation; Methodology; Supervision; Visualization; Writing–Original draft; Writing–Review & editing.

## Notes

^1^ In rare cases where a state-input combination never appeared in the learning phase, we allocated equal probabilities to all four subsequent states.^2^ Most of the excluded participants were in the *hard* FSM conditions: 13 in the explanation condition, eight in the prediction condition, and one in the control condition. In the *easy* FSM, three more participants did not meet the accuracy threshold (all of them were in the explanation condition).^3^ We used the final attempts only to calculate the accuracy in the feedback learning phase.
